# Fracture Resistance of Extensively Compromised Anterior Teeth Restored With Fiberglass Posts and Biomimetic Protocols: An In Vitro Study

**DOI:** 10.1111/jerd.70091

**Published:** 2025-12-24

**Authors:** Chiu Tzyy Haur, Emanuel Ewerton Mendonça Vasconcelos, Natália Gomes de Oliveira, Gabriela Queiroz de Melo Monteiro, Luís Felipe Espíndola‐Castro

**Affiliations:** ^1^ School of Dentistry, Universidade Federal de Pernambuco Recife Pernambuco Brazil; ^2^ School of Dentistry Universidade de Pernambuco Recife Pernambuco Brazil

**Keywords:** biomimetic, fiberglass post, polyethylene fiber, resin composite

## Abstract

**Objective:**

Polyethylene fiber (PF) and fiberglass (FG) posts have been recommended for intraradicular anchorage when there is a significant loss of coronal structure. However, few studies have evaluated their performance in anterior teeth combined with direct restorations as an alternative to single crowns. The objective of this study is to compare the fracture resistance and failure modes of endodontically treated anterior teeth restored with a FG post, a biomimetic technique using PF, and a direct resin composite (RC).

**Materials and Methods:**

Forty sound upper central incisors were selected, endodontically treated, standardized for clinical crown height, and randomly distributed into four groups (*n* = 10): Positive control: teeth with an intact clinical crown, subjected only to access and endodontic treatment (CT), RC, PF, and FG post. Restorations were performed according to specific clinical protocols using a universal adhesive system. Fracture resistance was tested on a universal testing machine under compressive load at a 45° angle (1 mm/min). Fracture patterns were analyzed under a stereomicroscope and classified as either repairable or catastrophic. Data were analyzed using ANOVA and Fisher's exact test (*α* = 0.05).

**Results:**

The CT group exhibited the highest mean resistance (840 N), followed by PF (740 N), FG (700 N), and RC (480 N), with a significant difference between the RC group and the others (*p* < 0.001). No significant difference was found among the groups regarding the failure mode (*p* > 0.05).

**Conclusion:**

FGs and PFs provided greater fracture resistance and a higher incidence of repairable fractures, with more favorable outcomes than when the procedure is performed exclusively with RC restorations.

## 1 Introduction

Endodontic treatment is indicated when the dental pulp is inflamed (pulpitis), infected (pulpal necrosis), or when there is an indication for elective treatment [1]. In these situations, teeth may exhibit reduced resistance due to carious lesions or trauma that necessitated treatment, as well as to the coronal access required for the endodontic procedure itself [2, 3, 4]. In this context, restoring teeth with significant coronal compromise associated with endodontic treatment is challenging [3, 5]. To promote the retention of the restorative material, intraradicular posts have been proposed [6].

Fiberglass (FG) posts, compared to cast metal cores, offer better esthetics, high tensile strength, and a modulus of elasticity similar to dentin, along with easy handling and good cost‐effectiveness [6, 7, 8]. Due to their anisotropic properties, FG posts can exhibit flexibility under radial loads, which can prevent stress concentration and promote a more favorable distribution of incisal forces to other areas of the tooth [6, 9, 10].

However, the use of fiber posts can present certain disadvantages, such as excessive removal of radicular dentin through the use of specific drills, the potential development of cracks or perforations during post space preparation, and, in the event of requiring endodontic retreatment, the removal of the post is highly challenging, as they are cemented with adhesive materials that have a strong bond to radicular dentin [7, 8, 11].

Recently, more conservative approaches, as alternatives to FG posts, have been proposed using biomimetic protocols. This word derives from the Latin “bios,” meaning life, while “mimesis” is linked to imitation. That is, these treatments aim to mimic the mechanical behavior of a natural tooth by enhancing adhesiveness [12, 13]. This therapeutic approach employs flowable resin composites (RCs), high‐molecular‐weight polyethylene or glass fibers, and 1‐mm horizontal increments of RCs [14].

Consequently, this approach is more conservative and has a lower risk of failure because it eliminates post space preparation and the insertion of posts inside the canal, allowing adhesion to occur in the cervical third and coronal dentin [7]. Nevertheless, for anterior teeth, where applied occlusal loads act on their palatal surface with shearing movements, there is a tendency for the clinical crown to flex in the cervical region [10]. Given this, these teeth may be more susceptible to fracture compared to posterior teeth [15, 16].

To minimize the risk of fracture, it has been suggested that a ferrule design preserving 2 mm of coronal structure may increase mechanical strength and reduce the incidence of catastrophic fractures [17, 18]. However, these findings remain inconclusive in the literature, as other studies indicate that post restorations without a ferrule can also be effective for the rehabilitation of extensively compromised teeth [19, 20].

Considering the above, the present study aimed to evaluate the fracture resistance of endodontically treated teeth restored with FG posts, biomimetic protocols, and conventional direct RC restorations. It also sought to measure compressive strength under different protocols using a universal testing machine and to evaluate failure mode using a stereomicroscope. The null hypotheses considered were: (I) there is no difference in the mechanical resistance of the teeth restored with the different protocols, and (II) there is no difference in the failure mode among the various protocols tested.

## 2 Materials and Methods

### 2.1 Study Design

This was an in vitro laboratory study. The present research was conducted in accordance with the Helsinki Declaration. Before its initiation, the study was submitted to and approved by the Human Research Ethics Committee (Universidade Federal de Pernambuco), Approval Number: 7.226.155.

### 2.2 Sample Preparation and Sample Size Calculation

For the sample size calculation, a previous study that also evaluated the fracture resistance of endodontically treated teeth restored with different restorative protocols was considered [21]. To observe statistically significant differences in fracture resistance between the groups, a change of 377.04 N was required [21]. Based on a mean standard deviation of ±231.09 N found in the study, a 5% significance level, and a test power of 80%, the minimum sample size per group was determined to be 8. Given potential losses during the analysis, the sample size was increased by 20% to *n* = 10 per group. The sample size was calculated for a comparison of more than 2 means with independent groups (analysis of variance [ANOVA]) using the website: http://estatistica.bauru.usp.br/calculoamostral/calculos.php (University of São Paulo, Bauru, São Paulo, Brazil).

For the present study, 40 intact, sound, and crack‐free maxillary central incisors, along with teeth obtained for therapeutic reasons (periodontal disease), were included. Teeth with radicular wear, those that did not present at least 1/3 of the cervical portion of the clinical crown intact, and teeth with a root length of less than 10 mm measured from the root apex to the cementoenamel junction (CEJ) were excluded.

The donated teeth were disinfected in a 0.5% chloramine‐T solution at 4°C for 1 week (ISO 11405:2003) and stored in distilled water under refrigeration at 4°C. The teeth were cleaned with periodontal curettes, followed by prophylaxis with a pumice/water slurry and a Robinson brush at low speed. Subsequently, the teeth were inspected under a stereomicroscope (Discovery V12, Zeiss, Germany) at ×40 to analyze the integrity of their surfaces. Teeth that exhibited fissures or cracks were excluded.

### 2.3 Endodontic Treatment and Sample Standardization

For endodontic treatment, a #1014 diamond bur (KG Sorensen, São Paulo, Brazil) was used to create the access opening, and an Endo‐Z bur (KG Sorensen, São Paulo, Brazil) to develop divergent walls. Subsequently, a size 15 K‐file (Univy Sense, Universo Odonto, São Paulo, Brazil) was introduced into the root canal until the apical foramen was visible. The length of the file was measured from the reference point on the cutting surface to the apical end, and the working length was established by subtracting 1 mm from this value. The canals were then prepared using only a #50.04 25 mm reciprocating file (Univy Sense, Universo Odonto, São Paulo, Brazil) driven by an endodontic micromotor (X‐smart, Dentsply, Ref A 1004, Switzerland), at a rotational speed of 500 rpm and 2 N of torque. For the canal preparation, it was established that for every two teeth instrumented, one #50.04 reciprocating file would be used, totaling 5 blisters containing four files for the treatment of the 40 teeth. Canal irrigation was performed with a 2.5% sodium hypochlorite solution (Rioquímica, São Paulo, Brazil). This was followed by a 1‐min rinse with a 17% EDTA solution (Biodinâmica, Paraná, Brazil), another minute with sodium hypochlorite, and a final rinse with 0.9% saline solution.

The root canals were dried using absorbent paper points. A eugenol‐free epoxy amine resin sealer (AH Plus sealer; Dentsply DeTrey, Konstanz, Germany) was applied around the canals up to the working length with a #10 K‐file (Univy Sense, Universo Odonto, São Paulo, Brazil). Then, the 50.04 gutta‐percha cone (Univy Sense, Universo Odonto, São Paulo, Brazil) was inserted 0.5 mm short of the working length to ensure good retention. The canal obturation was completed with the warm vertical compaction technique. A new periapical radiograph was taken to ensure the quality of the obturation. Finally, the canal orifices and root apices were sealed with modeling wax, and the teeth were kept in distilled water at room temperature for 72 h.

After endodontic treatment, the teeth were randomly allocated into the four groups, as detailed in Table 1. Randomization was performed using the website https://www.sealedenvelope.com/.

**TABLE 1:** Restorative protocols.

Groups	Restorative protocols
CT group: control group	The teeth underwent endodontic treatment only. No preparation was performed on the clinical crowns, leaving only the endodontic access cavity.
RC group: resin composite restoration	Six millimeters of obturating material were removed from the cervical region using Gates‐Glidden and Largo drills at low speed. Prophylaxis was performed with a pumice and water slurry. The tooth structure was then etched with 37% phosphoric acid (Angelus, Paraná, Brazil) for 30 s on enamel and 15 s on dentin, rinsed with an air/water spray, and dried. Subsequently, Scotchbond Universal Plus adhesive (Solventum, Minnesota, USA) was applied according to the manufacturer's instructions and light‐cured for 20 s (1200 mW/cm^2^, Grand Valo/Ultradent, Utah, USA). The resin composite restoration (Filtek Z350XT/Solventum, Minnesota, USA) was placed using an incremental technique, with each increment being light‐cured.
PF group: polyethylene fiber restoration	The post space preparation and adhesive system application followed the same protocol as the RC group. For the restorative procedure, a 1‐mm‐thin layer of flowable resin composite (Filtek Bulk Fill Flow/Solventum, Minnesota, USA) was applied, followed by a 0.35‐mm‐thick and 3‐mm‐wide polyethylene fiber (Ribbond, Oraltech, Paraná, Brazil) treated with Silane (Angelus, Paraná, Brazil) and adhesive (Scotchbond Universal Plus). The 14‐mm‐long fiber was fixed parallel to the long axis of the tooth in the deepest portion of the prepared space, leaving 5 mm of the fiber exposed for the clinical crown fabrication. The resin composite restoration was performed following the same protocol described for the RC group.
FG group: fiberglass post restoration	Two‐thirds of the root canal length was prepared using Gates‐Glidden and Largo drills (KG Sorensen, São Paulo, Brazil) at low speed, preserving a minimum of 3 mm of apical obturating material. To standardize the canal, a 1.0 post space drill (Angelus, Paraná, Brazil) was used. Prophylaxis of the canal was performed with a pumice and water slurry, and a fiberglass post (Exacto, Angelus, Paraná, Brazil) was placed, leaving 5 mm of the post exposed for the restoration fabrication. The post was treated with 70% alcohol, silane (Angelus), and adhesive (Scotchbond Universal Plus). Only the clinical crown was treated with the adhesive system (37% phosphoric acid, followed by an air/water spray, drying, and adhesive application). Luting was performed with a dual‐cure resin cement U200 (Solventum, Minnesota, USA). The clinical crown restoration followed the same protocol as described for the previous groups.

**FIGURE 1:**
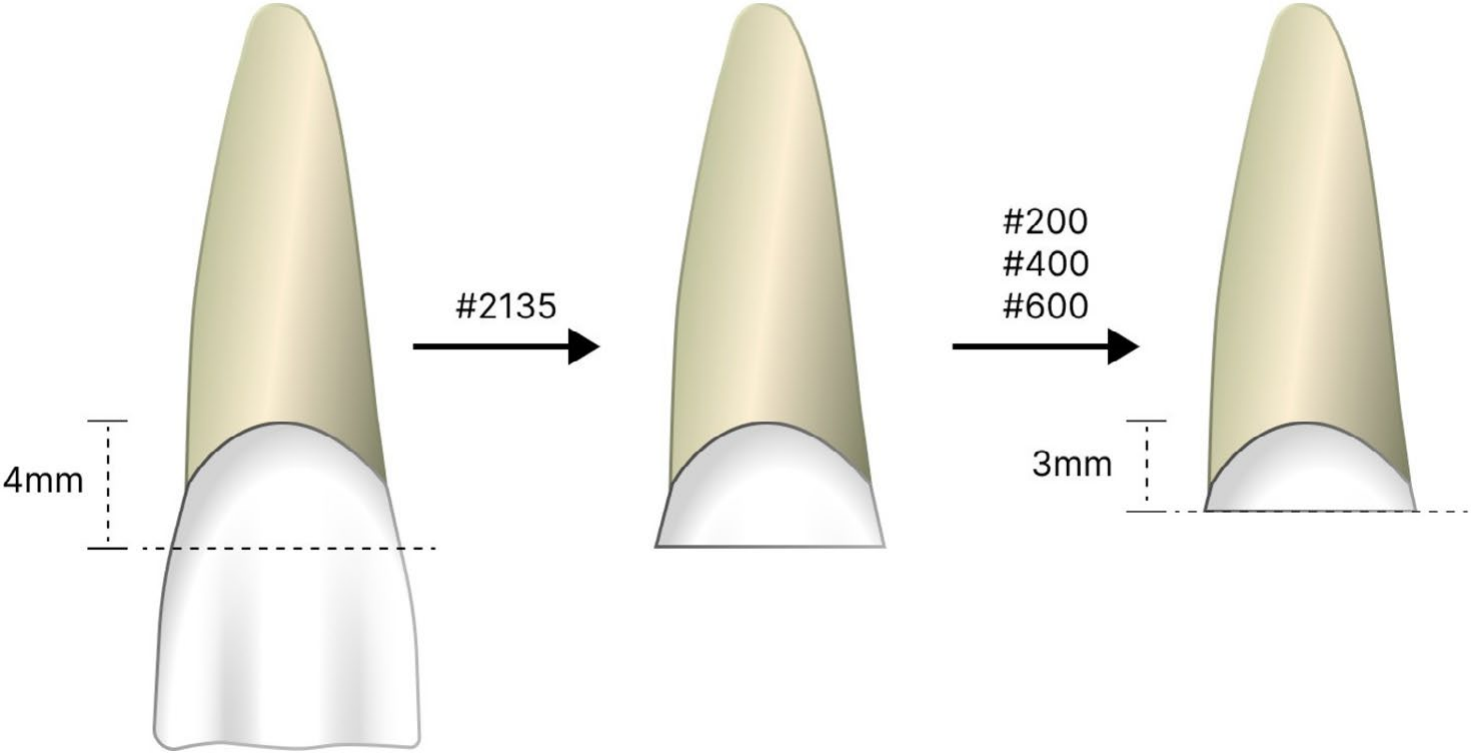
Standardization of the samples.

After the randomization, the teeth were sectioned transversally with a #2135 truncated conical diamond bur at high speed under refrigeration (KG Sorensen, São Paulo, Brazil), 4 mm above the CEJ (except for the control group, in which this wear was not performed). Following this step, the samples were ground on a metallographic polishing machine (Fortel, São Paulo, Brazil), with #200, #400, and #600 grit water‐cooled sandpaper to standardize the clinical crown to 3 mm, as illustrated in Figure 1 (without ferrule design).

Subsequently, the samples were embedded in polyvinyl chloride (PVC) pipe cylinders with chemically activated acrylic resin (Vipi Flash, VIPI, Rio Grande do Sul, Brazil) such that they were positioned centrally and in a vertical orientation using a delineator/parallelometer (Bio‐Art, São Paulo, Brazil) [22], without simulation of the periodontal ligament.

### 2.4 Restorative Protocols

**FIGURE 2:**
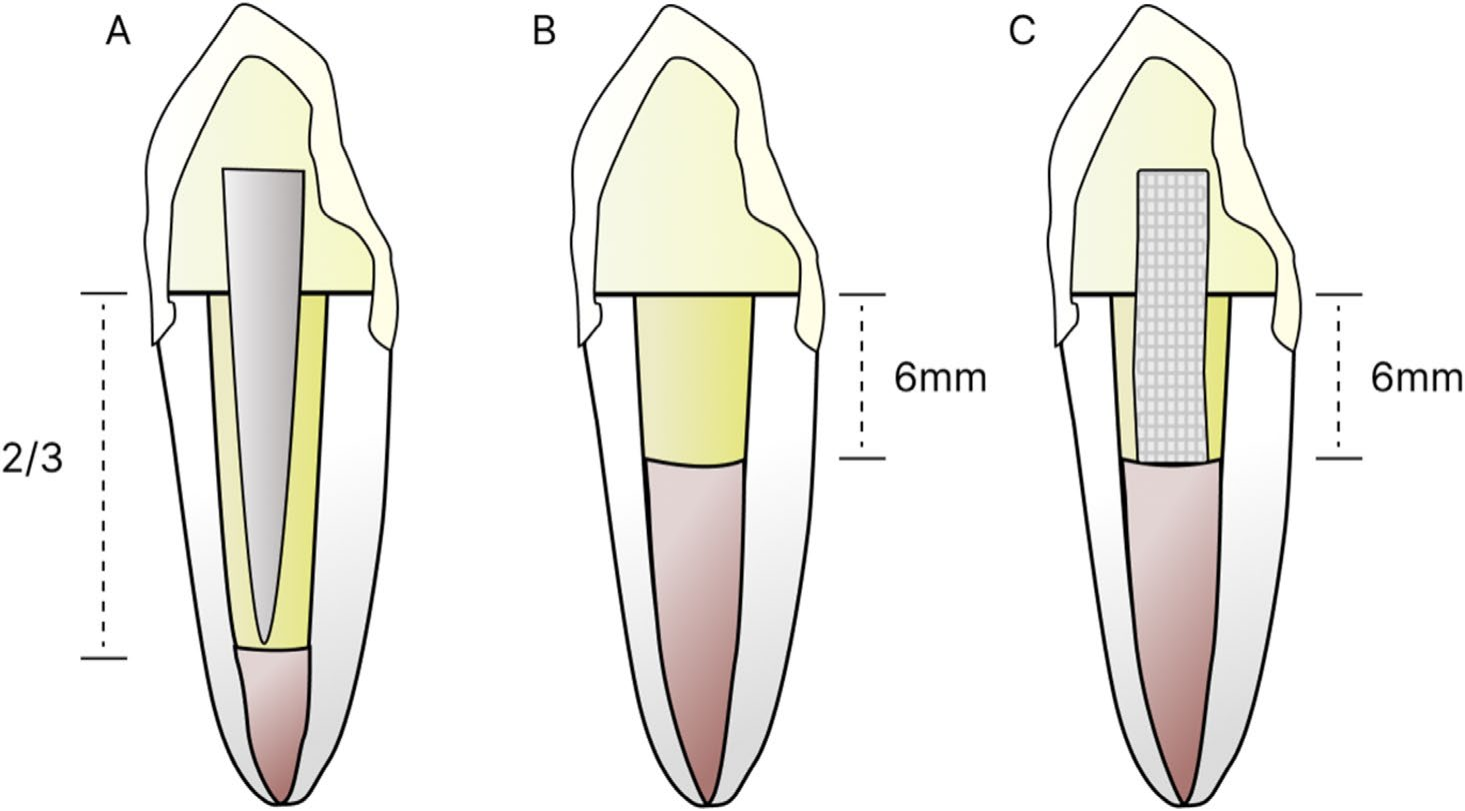
Tooth standardization by group. (A) FG; (B) RC; and (C) PF.

The treatment protocols are described in Table 1, and the tooth standardization is illustrated in Figure 2.

The materials used in the restorative protocols were summarized in Table 2.

**TABLE 2:** Materials used in the study and their respective compositions.

Material	Trade name and manufacturer	Composition	Batch number
Polyethylene fiber	Ribbond (Oraltech, Paraná, Brazil)	Ultra‐high molecular weight polyethylene fibers with an interlaced weave.	+D758O0/$$7339/16D20240821/T
Fiberglass post	Exacto (Angelus, Paraná, Brazil)	Fiberglass (80%) and epoxy resin (20%) by weight.	77083
Nanoparticle resin composite	Z350 XT (Solventum, Minnesota, EUA)	*Organic filler*: Bis‐GMA, UDMA, PEGDMA, and Bis‐EMA6, pigments. *Inorganic filler*: A combination of 20 nm silica filler and 4–11 nm zirconia filler (72.5% by weight and 55.6% by volume).	2224300320
Bulk fill flowable resin composite.	Filtek Bulk Fill Flowable (Solventum, Minnesota, EUA)	*Organic fillers*: Bis‐GMA, UDMA, Bis‐EMA6, TEGDMA, and Procrylat resins Inorganic fillers: a combination of zirconia/silica with a particle size range of 0.01–3.5 μm and ytterbium trifluoride filler with a range of particle sizes from 0.1 to 5.0 μm (64.5% by weight and 42.5% by volume).	2510700685
Dental adhesive	Scotchbond Universal Plus adhesive (Solventum, Minnesota, USA)	*Organic filler*s: MDP phosphate monomer, HEMA, Bis‐GMA, 3 M Vitrebond copolymer, ethanol, water, camphorquinone, silane, *Inorganic filler*: sillica	11060724
Dual‐cured resin cement	RelyX U200 automix (Solventum, Minnesota, USA)	*Base paste*: Silane‐treated glass powder, 2 propenoic acid, 2 methyl, 3‐(trimethoxysilyl)propyl ester, TEG‐DMA, Silane‐treated sílica, fiberglass. *Catalyst paste*: Silane‐treated glass powder, Silane‐treated sílica, calcium hydroxide, sodium *p*‐toluenesulfinate, 1,12‐dodecane dimethacrylate, titanium dioxide, calcium salts, calcium hydroxide, 1‐benzyl‐5‐phenyl‐baric acid.	2432800117
Phosphoric acid	Phosphoric acid (Angelus, Paraná, Brazil)	Phosphoric acid 37%, blue dye and thickener.	0680824
Silane	Silano (Angelus, Paraná, Brazil)	Silane and ethanol	1180823

Abbreviations: Bis‐EMA, bisphenol A ethoxylated methacrylate; Bis‐GMA, bisphenol A glycidyl methacrylate; EGDMA, triethylene glycol dimethacrylate; HEMA, 2‐hydroxyethyl methacrylate; MDP, 10‐methacryloyloxydecyl dihydrogen phosphate; PEGDMA, poly(ethylene glycol) dimethacrylate; SiO_2_, silicon dioxide; TEG‐DMA, triethylene glycol dimethacrylate; UDMA, urethane dimethacrylate.

### 2.5 Fracture Resistance Analysis

**FIGURE 3:**
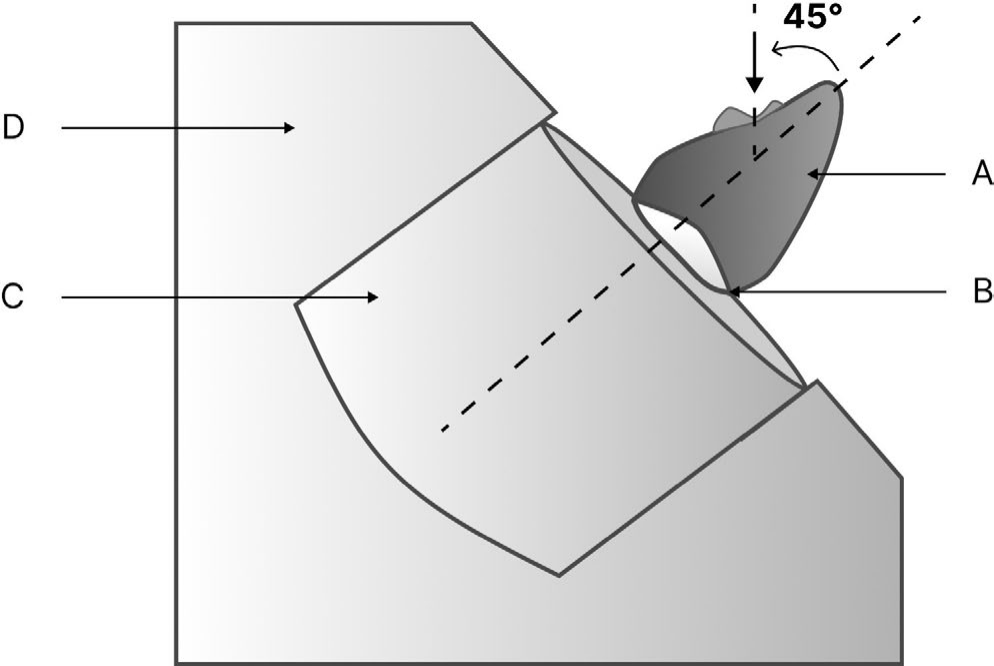
Mounting of the assembly to the base. (A) Resin composite clinical crown. (B) Acrylic resin block. (C) PVC cylinder. (D) Metallic base.

**FIGURE 4:**
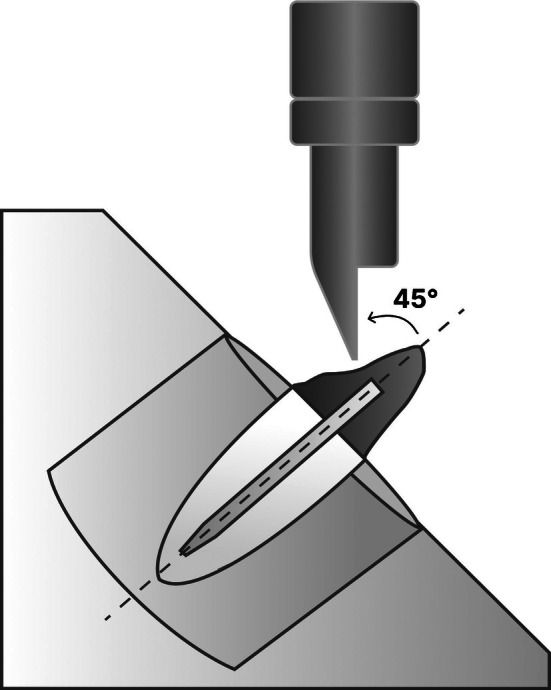
Diagram of a restored tooth in an acrylic resin cylinder.

The test was performed by adapting the sample‐acrylic resin assembly into a metallic jig with a central cylindrical opening. This jig was fixed to the base of a universal testing machine (OM150, Odeme Dental Research, Brazil), allowing the sample to be positioned at a 45‐degree angle. Subsequently, the tooth was subjected to a compressive load at a crosshead speed of 1 mm/min until fracture occurred (Figures 3 and 4). The maximum fracture resistance values were recorded in Newtons (N).

### 2.6 Failure Mode Analysis

**FIGURE 5:**
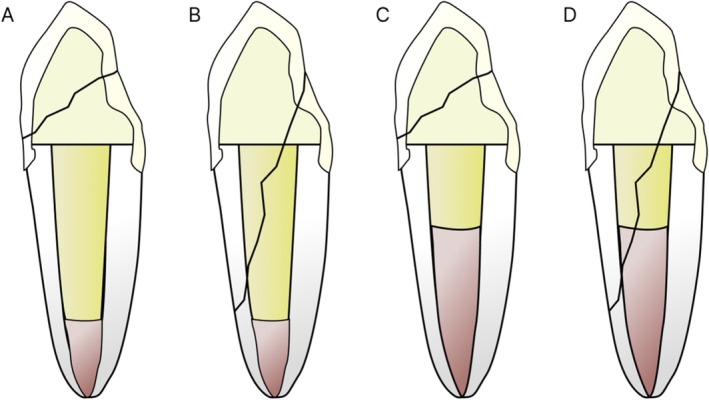
Representation of failure modes. (A and C) Repairable and (B and D) catastrophic fracture. Fractures above the CEJ were considered repairable.

The fracture pattern was evaluated using a stereomicroscope (Discovery V12, Zeiss, Germany), and image processing and capture were performed using the Zeiss ZEN 3.8 system. Subsequently, the samples in each group were classified as either repairable or catastrophic fractures. Repairable fractures were defined as those limited to the restoration or involving the tooth up to the CEJ. Oblique and longitudinal fractures below the CEJ were considered catastrophic (unrepairable) (Figure 5).

### 2.7 Statistical Analysis

The data were entered into an Excel spreadsheet and exported to SPSS software (version 17) for statistical calculations, with a significance level of 5%. ANOVA was used to investigate differences among the study groups. For more detailed information on the differences between specific means, Tamhane's multiple comparisons method was used. The data were analyzed descriptively using mean, standard deviation, and coefficient of variation for the numerical variable (fracture resistance) and absolute and percentage frequencies for the categorical variable (failure mode). For the comparison between groups, the *F* test (ANOVA) with Tamhane's multiple comparisons was used for the numerical variable, and Fisher's Exact Test was used for the categorical variable. Tamhane's post hoc test was chosen because the hypothesis of homogeneity of variances among the groups was rejected.

## 3 Results

Table 3 presents the means (in Newtons), standard deviations, and coefficients of variation for the fracture resistance by group. The lowest mean value was observed for the RC group (480 N), followed by the FG group (700 N) and the polyethylene fiber (PF) group (740 N). The control (CT) group had the highest mean (840 N). These results indicate a significant difference among the groups, confirmed by multiple comparison tests, which revealed a statistical difference between the RC group and all other groups. The variability was considered low, with coefficients of variation below 33.3% in all groups.

**TABLE 3:** Fracture resistance statistics (N) by group, showing mean and standard deviation (SD).

Group	Mean (SD)
CT (control)	840 (80)^(A)^
PF (polyethylene fiber restoration)	740 (140)^(A)^
FG (fiberglass post restoration)	700 (150)^(A)^
RC (resin composite restoration)	480 (150)^(B)^
*p*	*p* ^a^ < 0.001

*Note*: The presence of different superscript letters in parentheses indicates a significant difference between the respective groups.

^a^ *F* test (ANOVA) with Tamhane's multiple comparisons.

**FIGURE 6:**
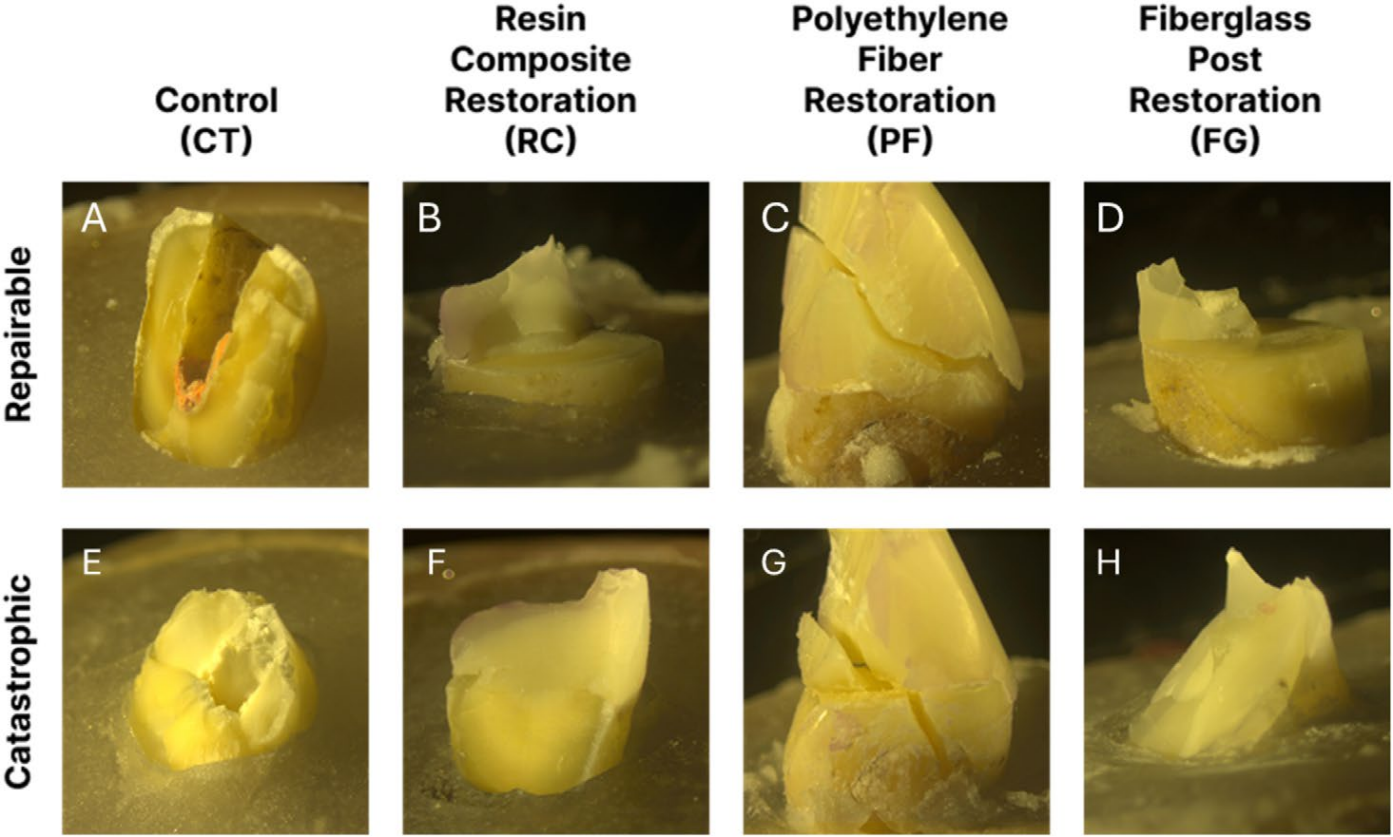
Classification of failure modes for the different groups. (A–D) Repairable fractures. (E–H) Catastrophic fractures. In the CT group, both repairable and catastrophic fractures occurred irregularly, without well‐defined fracture lines (A and E). In the RC group, repairable fractures predominantly occurred along the tooth–restoration interface (B). In the PF group, both repairable and catastrophic failures showed that the fractured fragment remained attached to the coronal remnant (C and G). In the FG group, repairable fractures exhibited a pattern similar to the RC group, with failures localized at the adhesive interface.

Table 4 presents the failure modes by group, with catastrophic fractures ranging from 40.0% to 50%, and the remainder being considered repairable. No significant difference was found among the groups (*p* > 0.05). Figure 6 illustrates the failure mode classification for the different groups using images of the fractured samples.

**TABLE 4:** Evaluation of failure mode by group (restored teeth).

Group (restored teeth)	Failure mode		
	Catastrophic, *n* (%)	Repairable, *n* (%)	Total, *n* (%)
FG (fiberglass post restoration)	4 (40.0)	6 (60.0)	10 (100)
PF (polyethylene fiber restoration)	5 (50.0)	5 (50.0)	10 (100)
RC (resin composite restoration)	4 (40.0)	6 (60.0)	10 (100)
CT (control)	4 (40.0)	6 (60.0)	10 (100)
*p*	*p* ^a^ = 1.000		

^a^ Fisher's exact test.

## 4 Discussion

The null hypotheses were partially accepted, as there was no statistical difference in the failure mode or reparability among the groups. Regarding fracture resistance, only the RC group had lower values when compared to the others (*p* < 0.001).

Restoring teeth that present significant structural compromise, whether from carious lesions, inadequate root canal preparation, or fractures, involves a complex procedure in which intraradicular posts may be used for its resolution [23]. However, concerning the FG post, its removal remains a challenge, as several factors must be considered, such as the type and size of the post, the luting cement used, the clinician's technical skill, and available resources [24], as well as the need for an atraumatic and conservative procedure, since removal can cause the appearance of cracks or fractures in the root and radicular perforation [25]. Thus, posts are not recommended for use in non‐circular, wide, or highly tapered canals, as these factors can compromise retention in the root canal by impairing adhesive resistance and promoting post displacement [26].

Moreover, the restoration of severely curved roots with rigid post systems may result in root canal perforations due to excessive dentin removal and root fractures caused by inadequate stress distribution [27]. PF, in turn, is a viable option due to its good strength, malleability, and esthetics [28]. Furthermore, the PF requires less canal gutta‐percha removal compared to the glass fiber post, as demonstrated in the methods and results of the present study.

No significant difference in fracture resistance was observed between the PF and FG groups. This finding corroborates the results of Behzadpour et al. [29], who reported that the fracture resistance values found were similar for the FG post and PF groups. This suggests that there was good load distribution along the surface and root canal, consequently leading to greater fracture resistance of the tooth, similar to that of a tooth without coronal destruction (CT group). The CT group was included in the present study to determine whether the direct restorative techniques tested could reproduce the biomechanical characteristics of intact teeth. The direct restorative procedures evaluated may serve as viable alternatives to more invasive and costly interventions, such as single‐unit ceramic crowns.

In addition to this study, a clinical trial that evaluated anterior teeth restorations reinforced with PF over a 3‐year follow‐up period reported no marginal irregularities, discoloration, changes in surface texture, loss of anatomical form, fractures, or retention failures [30]. In a recent systematic review comparing restorations of endodontically treated teeth with PF and prefabricated glass fiber posts, the authors observed lower fracture resistance in the PF groups. However, they emphasized that inconsistencies in the experimental methodology, such as variations in post brand, root dentin diameter, load application methods, storage medium, periodontal ligament simulation, ferrule effect, tooth type, crown positioning, and post length, make it difficult to draw definitive conclusions [31].

In this review, only four included studies evaluated fracture resistance in maxillary central incisors. Two of them found no significant differences between restorative protocols using PF and glass fiber posts. One study showed superior performance for glass fiber post restorations, while another reported better results for PF [31]. This divergence highlights the need for the present study to further investigate the clinical protocols proposed in the literature. The findings of the present study confirm that intraradicular reinforcements maintain mechanical strength. Therefore, restorations with PF and FG posts may be viable alternatives for extensively compromised anterior teeth. Furthermore, PFs reduce the removal of sound dental structure [32], demand a shorter length of material accommodated within the root canal [31], and consequently, can be more easily removed if a new endodontic intervention becomes necessary.

Clinical trials with follow‐up periods ranging from 5 to 10 years on endodontically treated teeth restored with FG posts have reported that the main failures are related to post fracture or endodontic complications [33, 34, 35]. These findings suggest that more conservative and reversible restorative approaches should be encouraged. According to Piovesan et al. [32], one significant advantage of PF–reinforced restorations is greater preservation of tooth structure, which helps protect against fractures under occlusal loading and improves clinical survival. Preserved dentin provides the solid structure necessary for the retention and support of dental restorations.

The restorative protocols tested in the present study are justified because maxillary anterior teeth are primarily subjected to non‐axial forces, which generate tensile stresses and increase the likelihood of mechanical failures, especially when restored with artificial crowns [17]. In contrast, posterior teeth typically receive compressive forces, which tend to be less damaging [17]. For this reason, FG posts are well indicated, as they present a reasonable stress distribution along the root due to their low modulus of elasticity, which is similar to dentin [16].

In the present study, there was no significant difference in the failure mode among the groups, including the CT group. The PF group showed a slightly higher rate of catastrophic fractures (50%) compared to the other groups (40%), as shown in Table 4. This type of fracture is classified as unfavorable because it precludes retreatment and jeopardizes the tooth's integrity [36]. This could be related to the shorter the post or the canal preparation, the less material volume is available to absorb forces. This limitation reduces the system's ability to dissipate stress, resulting in greater load transfer to the less rigid dentin, which increases the risk of radicular or catastrophic fractures [36]. Although no discrepant variations were observed, in the FG post group, repairable fractures occurred at the tooth/restoration interface (Figure 6). In the PF group, despite presenting a similar proportion of repairable fractures, these were associated with the fracture of the remaining coronal structure.

Another critical point to highlight is that, in the PF group, when a fracture occurred, the coronal fragment did not completely detach from the root remnant, as shown in Figure 6. Regardless of the failure mode, the fragment remained attached to the remaining root structure. This behavior can be considered a positive aspect, as it allows the patient to seek dental treatment without experiencing immediate anterior tooth loss.

The force exerted by anterior teeth in a healthy adult individual ranges from 120 to 240 N [37]. The study by Edmonds et al. [38] indicates greater progression in bite force with increasing age, with a value of up to 212.2 N obtained for anterior teeth. These values suggest that although the present study compared different restorative protocols, all tested techniques exhibit mechanical resistance well above that required during masticatory function. The findings of the present study demonstrated that reinforcement with PF and FG posts increased the mechanical resistance of the restorations without affecting the failure mode. These results suggest greater predictability and safety of these restorative approaches for anterior teeth.

The limitations of this study include the difficulty in standardizing teeth with varying lengths and thicknesses, and the fact that an in vitro study does not faithfully reflect the conditions in the intraoral environment or aspects of the stomatognathic system, such as temperature, humidity, and parafunctional habits. Furthermore, periodontal ligament simulation was not performed in this experimental model, which may limit the accuracy with which stress transmission during mastication can be reproduced.

## 5 Conclusion

Fracture resistance was higher in extensively compromised anterior teeth restored with either a FG post or a PF, showing performance comparable to the CT group and superior to the direct RC. The failure mode was similar among the groups, with a predominance of repairable fractures.

## Funding

This work was supported by Fundação de Amparo à Ciência e Tecnologia do Estado de Pernambuco (FACEPE), APQ‐1165‐4.02/24 and Coordenação de Aperfeiçoamento de Pessoal de Nível Superior—Brasil (CAPES)—Finance Code 001.

## Conflicts of Interest

The authors declare no conflicts of interest.

## Data Availability

The data that support the findings of this study are available on request from the corresponding author. The data are not publicly available due to privacy or ethical restrictions.
